# Comparative Analysis of Vitamin, Mineral Content, and Antioxidant Capacity in Cereals and Legumes and Influence of Thermal Process

**DOI:** 10.3390/plants13071037

**Published:** 2024-04-06

**Authors:** Corina Moisa, Anca Monica Brata, Iulia C. Muresan, Felicia Dragan, Ioana Ratiu, Oana Cadar, Anca Becze, Mihai Carbunar, Vlad Dumitru Brata, Alin Cristian Teusdea

**Affiliations:** 1 Department of Pharmacy, Medicine and Pharmacy Faculty, University of Oradea, 29 Nicolae Jiga Street, 410028 Oradea, Romania; corinamoisa@hotmail.com (C.M.); farmafeli@gmail.com (F.D.); 2 Department of Engineering of Food Products, Faculty of Environmental Protection, University of Oradea, 26 Gen. Magheru St., 410087 Oradea, Romania; 3 Department of Economic Sciences, University of Agricultural Sciences and Veterinary Medicine Cluj-Napoca, 3–5 Manastur Street, 400372 Cluj-Napoca, Romania; 4 Department of Medicine, Medicine and Pharmacy Faculty, University of Oradea, 1 University Street, 410087 Oradea, Romania; ratiu_ioana@yahoo.com; 5 INCDO INOE 2000, Research Institute for Analytical Instrumentation Subsidiary, 67 Donath Street, 400293 Cluj-Napoca, Romania; oana.cadar@icia.ro (O.C.); anca.becze@icia.ro (A.B.); 6 Faculty of Environmental Protection, University of Oradea, 26 Gen. Magheru St., 410087 Oradea, Romania; carbunar@yahoo.com (M.C.);; 7 Faculty of Medicine, “Iuliu Hatieganu” University of Medicine and Pharmacy, 400000 Cluj-Napoca, Romania; brata_vlad@yahoo.com

**Keywords:** cereals, minerals, vitamins, temperature, diet, health benefits

## Abstract

Cereals, as the world’s most consumed food, face challenges related to nutrient quality due to climate change and increased production impacting soil health. In this study, we investigated the vitamin and mineral content, polyphenols, and antioxidant activity in cereals from Western Romania, analyzing whole and hulled wheat, rye, oat, and soybeans before and after heat treatment. Samples from 2022 crops were processed into dough and subjected to 220 °C for 30 min. The results reveal that, despite efforts to optimize nutrient content, cereals, particularly after heat processing, exhibited lower vitamin and mineral levels than the recommended daily intake. The decrease in polyphenols and antioxidant capacity was notable, with rye flour experiencing the largest decline (15%). Mineral analysis showed copper levels in decorticated wheat decreased by 82.5%, while iron in rye decreased by 5.63%. Soy flour consistently displayed the highest calcium, magnesium, and potassium levels, whereas oat flour had the highest zinc and copper levels before and after heat processing. The study highlights the concerningly low vitamins and minerals contents in cereals, as well as in the final products reaching consumers in the Western part of Romania, and contributes to the assessment of measures that are meant to improve the contents of these minerals.

## 1. Introduction

Vitamins and minerals are a group of compounds essential for the proper functioning of the body, requiring a balanced and proper diet in order to ensure adequate concentrations in the body [1]. Studies show that in recent decades a diet rich in fruit, vegetables, meats, and whole grains that provide the body with the necessary vitamins and minerals for the proper function has been gradually replaced by a diet poor in micronutrients consisting mainly of processed meats, fats, processed grains and sugars [2]. In Romania, according to the National Study on the Prevalence of Diabetes, Prediabetes, Overweight, Obesity, Dyslipidemia, Hyperuricemia and Chronic Kidney Disease (PREDATORR), 31.4% of adults aged 20–79 suffer from obesity and 34.6% are overweight [3]. According to international studies, there is great concern because obesity is associated with an increased risk of metabolic, cardiovascular, and renal diseases, with childhood obesity increasing tenfold worldwide in the last four decades [4,5].

The daily requirement of vitamin and mineral intake depends on several parameters, such as gender, ethnicity, medication, drug and alcohol consumption, age, physical activity, and certain pathologies [5,6]. Deficiencies in vitamins and minerals resulting from inadequate dietary intake have been linked to neurological, cardiovascular, renal, and hormonal disorders, incurring additional costs to healthcare systems, and thus emerging as an important public health issue [7,8,9].

Cereals, comprising staples such as wheat, rye, oat, and soybeans, represent a foundational component of global diets, and serve as primary sources of essential vitamins and minerals. Rich in carbohydrates, fiber, and plant-based proteins, cereals contribute significantly to daily nutritional intake [10]. The productivity, quality, and composition of cereals are intrinsically linked to numerous factors, including the geographical position of crops—encompassing specific relief and climate considerations [11]; soil type and its natural composition, including mineral content and enzymology [12]; practices related to soil management, encompassing crop-specific agricultural tasks, crop rotation, irrigation, classical and modern fertilization treatments [13,14,15,16], and short/long-term soil monitoring [11].

These grains also house a diverse array of vital micronutrients, including various B-group vitamins, iron, zinc, magnesium, potassium, and phenolic compounds [17]. Phytic acid plays a crucial role, influencing the levels of minerals in cereals and the bioavailability of these minerals in the human body by forming insoluble salts (phytates) with magnesium, calcium, and iron, hindering their absorption [18].

Antioxidant compounds in cereals, predominantly phenolic compounds, play a vital role in combating various ailments, including cancer, diabetes, and cardiovascular diseases. Wheat bran phenolic compounds exhibit proven antimicrobial properties against several pathogens [19] and anti-inflammatory action in liver conditions by inhibiting cytokine production [20].

The dynamic of vitamin and mineral levels in cereals undergoes a significant transformation before and after processing. The processing of cereals, such as heat treatment, milling, and other technological steps, can have a profound impact on the nutritional composition of the final product. Studies investigating these changes have revealed that, despite efforts to optimize nutrient content, cereals, particularly after heat processing, may exhibit alterations in vitamin and mineral levels. Heat treatments, such as baking or cooking, can lead to a reduction in the concentrations of certain vitamins and minerals, potentially affecting their bioavailability [21,22]. The enhancement of vitamin and mineral levels in bread has been achieved by incorporating rice flour, increasing the quantities of zinc, iron, potassium, phosphorus, and niacin by 10% [23]. Improving the nutritional quality of bread remains a priority, with studies exploring the impact of processes such as boiling, irradiation, decortication, fermentation, germination, and storage on vitamin and mineral content [24].

Considering that vitamins and minerals are essential for the optimal functioning of the entire body and that cereals are at the bottom of the food pyramid, being the most consumed food, the aim of this study was to determine the contents and levels of several vitamins and minerals before and after temperature processing in cereals harvested from a certain area in Western Romania. The importance of the study lies in the fact that cereals, and therefore bread, are some of the most consumed food products worldwide, and play an important part in human nutrition. Thus, the contents of minerals and vitamins and the dynamics of their concentration are crucial in order to achieve a balanced diet and nutritional state.

## 2. Results

### 2.1. Temperature Treatment on Mineral Content

The mineral content showed significant variations (Table 1). The highest level of Ca was found in P4, followed by P5, and with a much lower level in P3 and P1. P2 was the sample with the lowest level of Ca. The highest level of Mg was found in P4, followed by P5, P3 and P1 at about half the level. P2 was also the sample with the lowest level of Mg. The highest level of Zn was found in P5 and P1, followed by P3 and P4. Again, P2 was the sample with the lowest level compared to the other samples. The highest level of Fe was found in P4 and P5, followed by P1 and P3. P2 was the sample with the lowest level of Fe. In the case of Cu, P5 and P4 were the samples with the highest level, followed by P3 and P1. P2 had a low level of Cu. The highest level of K was in P4 and, by a large margin, in P3, P5 and P1. As in the previous cases, P2 was the sample with the lowest level. Of the cations analyzed, the level of K was the highest (P4), followed by Mg (P4), Ca (P4), Fe (P4), Zn (P5) and Cu (P5).

Following heat processing, all samples showed a low level of minerals compared to the daily needs of the consumers (presented in Table 2, indicating the unfavorable action of heat treatment on the nutritional quality of the products obtained. 

**Figure 1:**
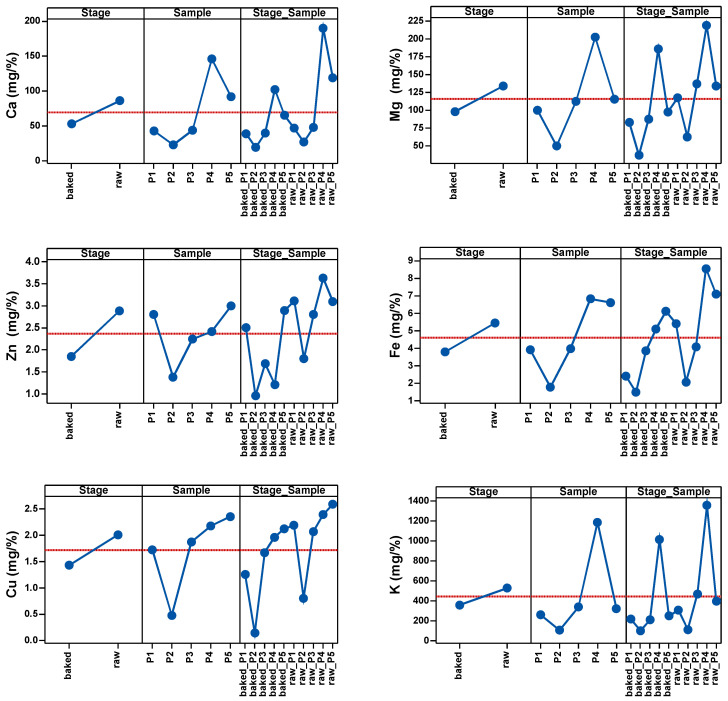
Main effects of Stage, Sample and Stage_Sample factor on the cation content.

As shown in Table 3 and Figure 1, the most significant changes in mineral levels were observed in Cu, K, Fe and Zn. The highest influence of temperature on the samples was observed in Cu and the lowest in Fe.

Therefore, before heat treatment, the values of P4 and P5 were above the average of the samples, and after heat treatment P4 was the only sample with above-average values.

In the case of Mg, only P2 had values below the average before heat treatment. The same was observed for Zn^2+^ before heat treatment. In the case of Fe, only P4 and P5 had values above the average values. In the case of Cu, only P2 had below-average values before heat treatment. In the case of K, only P1 and P2 had below average values before heat exposure.

After the heat treatment of the five samples, P4 remained above the sample average with the highest Ca level, followed by P5 at the average level of the sample. Ca level in the other samples was low and below the average of the determinations. When it comes to Mg, P4 was the sample with the highest Mg level, P5 was just below the sample average, and P3, P1 and P2 had low Mg levels. Low levels of Zn were observed across all samples, with P1 and P5 being the samples with an above-average level of Zn. Only P4 and P5 had Fe levels above the average of the determinations. P4 and P5 had above-average levels of Cu; P3 was at the average level and P1 and P2 were below the average. P4 remained the sample with the highest K level after heat treatment.

Additionally, in order to ensure a standardized comparison that accurately reflects the adequate composition of the analyzed samples, the moisture content of the samples has also been taken into account, and the data on a dry weight basis were recalculated as well, as presented in Table 4. Across the various samples, notable variations in nutrient levels are evident, reflecting the impact of baking on the nutritional composition of the samples. For instance, in raw samples, Ca content ranged from 30.30 mg per 100 g of dry weight in P2 to 204.60 mg per 100 g of dry weight in P4. Similarly, Mg concentrations varied as well, with P2 exhibiting the lowest concentration at 70.95 mg per 100 g of sample dry weight, while P4 displayed the highest concentration at 235.59 mg per 100 g.

Comparing the raw and baked samples, there was a noticeable change in Ca content in baked samples, especially when considering raw and baked P4, while increased K concentrations have been recorded for P1 and P4. Moreover, Fe saw a significant increase in sample P5, from 7.57 mg per 100 g of dry weight to 10.07 mg per 100 g of dry weight.

These changes indicate how temperature treatment affects moisture levels and mineral content, showing how nutrient availability and concentrations can change due to heat and thermal processes taking place.

#### Multivariate Analysis

**Figure 2:**
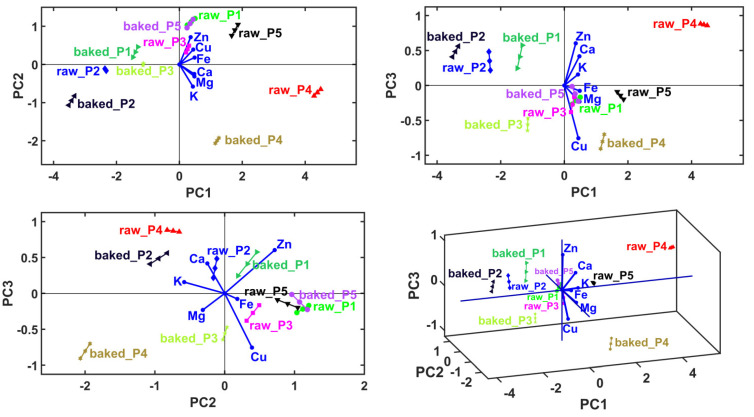
Principal component analysis (PCA) biplots represented in 2D and 3D views, with first three principal axes, PC1–PC3.

The main objective of the multivariate analysis was to determine the sample clusters, and for each sample, which are the cations with the dominant level. From the mentioned multivariate analysis sequence, only the MANOVA yielded statistically significant results (*p* = 0.05), thus the use of this method gives 95% accuracy to the overall sample clustering process. The results of PCA are presented in Table 5 and Figure 2. According to the Kaiser–Guttman rule [25], considering only the principal components with eigenvalues greater than 1, we derived a cumulative variance value of 77.02%, which only implies PC1. A total explained variance higher than 95% (which can assure high accuracy) was achieved by considering at least the first eight principal components (PC1–PC3 gives 96.77% of explained cumulative variance, Table 5). This situation forced the use of linear discriminant analysis (LDA) in conjunction with the MANOVA (*p* = 0.05) method to generate the correct number and content of sample clusters, and finally, graphical verification with the HCA method.

The first figures are the PCA biplots overlaying the sample groups with principal coordinates with the variable vectors. The variable vectors start in the biplot origin and point out the areas that prescribe the highest levels of the corresponding variable (i.e., cations level). Accordingly, the neighboring areas have the lowest levels of the corresponding variable in the opposite direction of the vectors. Accordingly, the PCA method generates a qualitative relative comparison between the samples. Also, it provides information about the dominant variables that characterize the samples. The last figure shows variable grouping based on their correlation (in principal coordinates) prescribed graphically by small solid angles between their corresponding vectors.

In the case of P1, a reduced mineral content in Fe^2+^, followed by Cu, Mg, K, Zn and Ca, was observed. In the case of P2, a reduced mineral content in Cu, followed by Zn, Mg, Ca, Fe and K, was observed. In the case of P3, a reduced mineral content in K, followed by Zn, Mg, Cu, Ca and Fe, was recorded. At P4, a reduced mineral content in Zn was showed, followed by Ca, Fe, K, Cu and Mg. At P5, a reduced mineral content in Ca was observed, followed by K, Mg, Fe, Cu and Zn.

**Figure 3:**
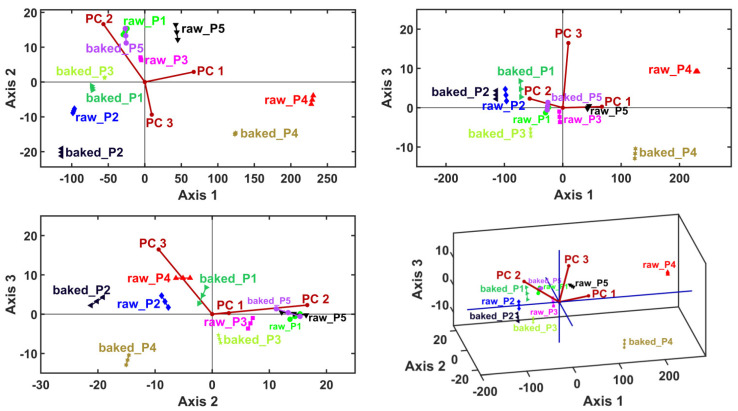
Linear discriminant analysis (LDA) biplots represented in 2D and 3D views, with the first three canonical axes, Axis1, Axis2 and Axis3.

Clustering information was generated by considering the sample principal coordinates (i.e., PCA scores) as input data for LDA, MANOVA (*p* = 0.05) and HCA. Linear discriminant analysis generates canonical coordinates for the samples (i.e., LDA scores) and variable vectors (PC1–PC6) (i.e., LDA loadings). This multivariate method calculates the canonical coordinates to maximize the relative distances between samples—thus, it is expected to generate the proper sample clusters (Table 6 and Figure 3).

**Figure 4:**
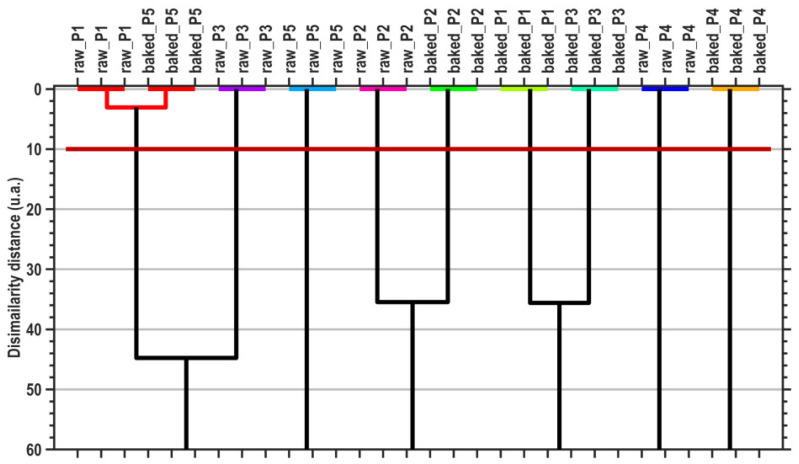
Dendrogram graphical representation of hierarchical cluster analysis (HCA). The horizontal dark-red line denotes the dissimilarity threshold distance that generates the proper cluster number, generated by the MANOVA (*p* = 0.05) multivariate method.

The canonical coordinates are also used by the MANOVA (*p* = 0.05) method to generate pairwise sample comparisons. Table 7 presents the statistical significances (*p*-values) of the pairwise sample comparisons, emphasizing the proper cluster number and content that are displayed in Figure 4 (graphical verification with the HCA method).

Since the assumption was that the cereals grown and harvested in the Western regions of Romania were processed and consumed only in this area, the mineral content in white bread purchased from a supermarket in the area when conducting the research was also determined, this being the most widely consumed product by the population. The results are presented in Table 8 and are very close to the results for the analyzed cereals in the current study.

### 2.2. Temperature Impact on Polyphenols, Vitamins Content, and Antioxidant Capacity

The total polyphenolic content, as well as the antioxidant capacity, were further measured, and the results are illustrated in Table 9 and Table 10. Three measurements were conducted for each sample, and the results show that the total polyphenol concentrations, as well as the antioxidant capacity, remained similar after thermal processing. The highest polyphenolic counts were recorded in P1 and P5, both before and after thermal processing, directly correlating with their antioxidative properties.

Integral wheat (P1) exhibited the highest total phenolic content and antioxidant capacity, with 1090 µg GAE/g and 2.307855 µg AAE/g, respectively. Soy (P4) showed the lowest total phenolic content and antioxidant capacity, with 96 µg GAE/g and 0.21085 µg AAE/g, respectively. Whole grains, like integral wheat, typically have higher phenolic contents and antioxidant activities than refined grains, such as dehulled wheat.

The results of the determination of vitamin levels in the studied cereals are presented in Table 11. The major vitamin deficiency is worrying considering the role these vitamins play in the functioning of the body and the importance of ensuring optimal vitamin requirements in the diet.

When analyzing the vitamin contents of the five samples before thermal processing, no vitamin except B3 reached adequate concentrations over the quantification limit (LQ) in order to be deemed appropriate for further measurements. Thus, the samples were not further assessed or measured after thermal processing, as the vitamin content was already too low and unsatisfactory, as illustrated in Table 11.

The results of the analysis of this product indicate that the mineral content of the final products derived from cereals consumed by the population of Western Romania is low compared to the contents obtained in the determinations of cereals harvested from the same area. The low mineral content combined with the high consumption of cereal products raises concerns about the health status of the population in all age groups.

## 3. Materials and Methods

### 3.1. Cereal Selection

The studied cereals were selected based on the types of cereals cultivated in the area of Săcuieni town, Bihor County, Romania, situated at 47.35° latitude and 22.1° longitude. The soil in the experimental area is slightly acidic, with a pH between 5.95 and 6.40, low humus concentration, a low total nitrogen content of 0.0075, and low and very low mobile phosphorus and potassium contents, of 12 and 60 ppm, respectively.

Five types of flour were included in our study, namely, whole wheat, hulled wheat, rye, soy, and oat. This has mainly been done because different cereals exhibit distinct nutritional profiles, containing varying concentrations of vitamins and minerals. The cereal samples were obtained from crops grown in 2022 in a conventional system on a sandy, medium-coarse-textured soil (psalmosol class). All grains (wheat, rye, soybean, and oats) were milled using an electric robot. The wheat was hulled using a dislocator (wheat finisher). A part of the resulting flour was analyzed as such, and the other part was used to obtain a dough with a soft consistency. The dough was prepared from 100 g flour and 60 g distilled water, and was subjected to a temperature of 220 °C for 30 min in an electric oven. The properties of the samples are shown in Table 12.

Determinations were performed on the flour samples obtained from cereals, and these results were correlated with the bread samples obtained from the same cereals after the flour was transformed into dough and then subjected to heat processing.

The results obtained were analyzed comparatively to determine the effects of heat treatment on mineral content. Since the aim of the study was not to produce a product with a high acceptance among the population, further studies on sensory analysis, the rheological parameters of the dough, and the physicochemical and textural properties of the obtained preparation were not deemed necessary.

### 3.2. Chemicals and Reagents

Methanol (≥99.8%) and acetic acid (≥99.8%) used for high-performance liquid chromatography (HPLC) were purchased from VWR Chemicals (Solon, OH, USA). All other standards (Thiamine hydrochloride, Riboflavin, Niacinamide, Pyridoxine hydrochloride, Cyanocobalamin) used were from Sigma-Aldrich (Steinheim am Albuch, Germany). HNO_3_ 65%, H_2_O_2_ 30% and ICP multi-element standard solution IV, 1000 mg/L, were purchased from Merck (Darmstadt, Germany). Ultrapure water from an Ultraclear Evoqua purification system (Erlanger, Kentucky, AL, USA) was used to prepare the standard solutions and dilute the samples. All solvents were HPLC grade from VWR, and the ultra-pure water was obtained using the ULTRACLEAR UV UF EVOQUA Purification system, Pittsburg, PA, USA. We used the ACW Kit from Analitk Jena (Jena, Germany). All other standards used were from Sigma-Aldrich.

### 3.3. Determination of Vitamins B

The samples were further processed by extracting 0.250 g of sample with 1 mL MeOH. The samples were centrifuged using a Hettich D-78532 microcentrifuge (Tuttlingen, Germany) at 11,000 rpm for 2 min; the supernatant was filtered through a 0.45 µm cellulose filter and then analyzed by UHPLC Vanquisher H Dionex (Thermo Fisher Scientific, Dreieich, Germany) with a DAD detector for the analysis of thiamine (B1), riboflavin (B2), nicotinamide (B3), pyridoxine (B6) and cyanocobalamin (B12). The mobile phase was composed of ultra-pure H_2_O with 1% acetic acid and MeOH in gradient with a flow of 0.3 mL/min. The chromatographic column used was a AccucoreaQ 100 × 2.1 mm, 2.6 μm (Thermo Fisher, Waltham, MA, USA), kept at 25 °C. The injection volume was 8 µL and the detector was set at 270 nm.

### 3.4. Mineral Profile

The minerals (K, Ca, Mg, Fe, Cu and Zn) were determined using an inductively coupled plasma optical emission spectrometer Perkin Elmer Optima 5300DV (ICP-OES) (Waltham, MA, USA) after microwave-assisted digestion using a BerghofXpert system (Eningen, Germany). An amount of 500 mg of the sample was digested using 8 mL HNO_3_ 65% and 2 mL H_2_O_2_ 30% in a polytetrafluoroethylene digestion vessels, using a four-step digestion program (140, 170 and 190 °C—heating; 50 °C—cooling) for a total digestion time of 40 min. Afterward, the vessels were cooled down and the volume was made up with ultrapure water. Blanks were prepared in each lot of samples.

### 3.5. Moisture Content

The moisture contents of the flour and bread samples were determined using the AOAC’s official method 925.10 by drying at 105 °C until the resulting weight was constant [26].

### 3.6. Determination of Total Phenolic Content

Then, 5 mL of distilled water, 1.5 mL of sodium carbonate solution (10%), 0.5 mL of extracted sample and 0.5 mL of Folin–Ciocalteu solution where pipette into a 15 mL centrifuge vail. After the samples were kept for 45 min at room temperature in the dark, they were measured at a wavelength of 765 nm (Spectrum BX II, Perkin Elmer, USA). The results have been expressed in gallic acid equivalent. The samples were created in triplicate [27].

### 3.7. Determination of Total Antioxidant Capacity

After methanol extraction, the samples were directly injected into PHOTOCHEM (Analytik Jena, Germany) and the antioxidant capacity was measured suing the ACW kit and expressed in equivalent ascorbic acid. The samples were made in triplicate.

### 3.8. Determination of Vitamin D3 (Cholecalciferol), Vitamin A (Retinyl Acetate), Vitamin K (MK4, MK7)

Here, 0.250 g samples were extracted with 1 mL MeOH. The samples were centrifuged (Microcentrifuge Hettich D-78532, Germany) at 11,000 rpm for 2 min; the supernatant was filtered through a 0.45 µm cellulose filter and then analyzed by UHPLC Vanquisher H from Dionex, Thermo Fisher Scientific, Germany, with a DAD detector for the analysis of Cholecalciferol, Retinyl Acetate, MK4, MK7. The mobile phase was composed of ultrapure water (A) and methanol (B). The isocratic elution was performed at 1 mL/min in a proportion of 98% B. The chromatographic column used was a Acclaim C30 150 × 46 µm, 5 µm (Thermo Scientific, Sunnyvile, CA, USA), kept at 30 °C. The injection volume was 10 µL and the detector was set at 265 nm [28].

### 3.9. Statistical Analysis

The design of experiment (DOE) aimed to compare the levels of different cations in five different cereals (P1–P5). Thus, two factors were considered: the raw factor and the baked factor. Univariated statistical analysis consisted of two-way analysis of variance (ANOVA, *p* = 0.05) with a post-hoc multiple pairwise sample mean comparison test, established by Dunn–Sidak, with a confidence interval of 95% (*p* = 0.05).

Multivariate statistical analysis considered as samples the interaction factor when raw and baked. The variables consisted of the levels of different cations in five different cereals (P1–P5).

The multivariate approach included several methods: principal component analysis (PCA), linear discriminant analysis (LDA), multivariate ANOVA MANOVA (*p* = 0.05), and hierarchical cluster analysis (HCA). All these methods enable comparisons between multivariate data that consist of samples’ multivariate profiles. Each multivariate profile sequentially gathers all the parameter values for the sample. The multivariate analysis was performed and graphically designed with a custom-made application based on standardized procedures from MATLAB 2022b CWL (The MathWorks Inc., Natick, MA, USA) [24].

## 4. Discussion

According to the 2020 Global Nutrition Report, one-third of the population is obese or overweight. Bread is one of the most widely consumed foods, high in carbohydrates and fats, but unfortunately low in vitamins and minerals [5].

Wheat is one of the most widely used cereals in the diet, being an important source of vitamins and minerals and containing little fat. After wheat grain is processed into flour, its nutrient content changes through fermentation and heat treatment in the process of producing various bakery products. After the hulling of wheat, white flour is produced, which is lower in nutrients due to the refining process [29]. A constant concern, especially in developed countries, is to improve the quality of bread and cereal foods in general so as to ensure a healthy diet [30]. This phenomenon has been ever more evident during the past few years, when the general interest in plant-based diets has increased substantially [31]. The consumption of whole grain products is probably lower also due to their organoleptic properties; whole grain products being associated with lower acceptability [32]. In European countries, wheat is the staple food crop, providing up to 50% of the total energy intake, with other cereals and beans following behind [33]. The concentration of iron and zinc in the grain is directly influenced by the minerals available in the soil, and these minerals are found in the embryo and aleurone of the wheat grain, along with the B vitamins, calcium, and magnesium. The embryo and the aleurone are largely removed during hulling, which is why these minerals are present to a lesser extent in white flour [29,34].

Existing research has also confirmed the decrease in vitamin and mineral contents under the thermic treatment of cereals and legumes [35]. Thus, our findings were similar to those obtained by Barantama and Simard, who recorded a significant decrease in minerals and vitamins after processing common beans [36]. Additionally, studies have identified that some cooking methods involving temperature do not affect the content of Fe to such great extent as the concentrations of other minerals [37,38], and this aspect has also been confirmed by our findings, with the lowest impact of temperature being on Fe concentration, across all samples, with oat and soybean flours still remaining above average Fe concentrations. However, our analysis yielded different results when compared to some other studies, with Hemalatha et al. not recording any difference in the bioavailability of Zn in wheat after heat treatment [37].

Nevertheless, different combinations of wheat flour and flour from other cereals can be used to improve bread quality. Thus, studies show an increase in the levels of K, Mg, Na, Zn, Fe and polyphenols when combined with quinoa flour [39]. The quality of the bread was also improved by the addition of chickpea flour, which resulted in increased levels of Na, Mg, Fe and Zn, as well as phenolic compounds and flavonoids with antioxidant activity [40]. Furthermore, better antioxidant properties were obtained when combining amaranth with corn flour, while adding millet to corn flour yielded increased levels of Ca, Fe, Zn and fibers [41,42]. Higher protein levels were achieved by combining wheat flour with lentil flower or *Moringa olefeira* leaf powder, with the latter also increasing the levels of polyphenols and minerals, such as Ca, Zn and Fe [43,44].

Flour fortification is a crucial public health strategy aimed at enhancing the nutritional value of flour by adding essential micronutrients, such as vitamins and minerals. Typically, iron, folic acid, and various B vitamins, including thiamine, riboflavin, and niacin, are commonly incorporated into flour during the fortification process [45]. Thus, in Mexico, commercial wheat flour is fortified with folic acid, Fe and Zn, increasing the antioxidant capacity [46].

All the analyzed samples are present in the diet after heat processing. In order to limit the unfavorable influence of temperature on bioactive compounds and antioxidant phenolic acids, as well as to preserve endogenous nutrients and reduce the production of toxic reaction compounds, Tian et al. thermally processed wheat flour to produce bread and pancakes at temperatures below 100 °C [47]. The decrease in antioxidant capacity after heat processing was also demonstrated by Cammerata et al., who conducted studies on raw and boiled pasta obtained from wheat species [48]. Analyses of vitamin and mineral content and antioxidant capacity were also conducted by Furuichi et al. on various barley species. However, they did not determine the levels of these compounds after thermal processing in various food preparations [49].

Considering the differences in the quantities of vitamins and minerals in cereal flour (expressed per 100 g of the sample) and various types of food products (expressed per 100 g of the sample/bread) obtained after heat processing, we compared the two categories. This highlighted the fact that the population’s diet is deficient in vitamins, minerals, and antioxidants necessary for the optimal functioning of the body. These differences are also due to the fact that 100 g of flour does not yield 100 g of bread. The products most widely consumed by the population are those obtained from hulled wheat, which is low in these components.

The observed variations in mineral content and nutrient concentrations between raw and baked samples underscore the multifaceted nature of food processing and its impact on nutritional composition. Several factors contribute to these differences, including the effects of heat treatment, moisture loss, and biochemical transformations during baking. Heat exposure can lead to the degradation or loss of certain nutrients, particularly heat-sensitive vitamins and antioxidants, thereby influencing the overall nutrient profile of the baked samples. Additionally, moisture loss results in a concentration effect, leading to higher nutrient concentrations per unit dry weight. This phenomenon was better observed when assessing the moisture content of the samples and reporting the results of the measurements in the dry weight system, as increases in the concentration of certain minerals, such as K, were recorded.

Although the moisture content differs between flour samples compared to bread samples, and the results were reported per 100 g of product, both cereal-derived flour and bread are deficient in terms of mineral and vitamin content, as well as antioxidant capacity, relative to the daily requirements of the population. This is concerning as it predisposes the population at all ages to the development of various pathologies.

Phenolic compounds are mainly found in the bran and germ, which are removed during the refining process [50,51]. Polyphenolic compounds have been proven beneficial in various gastrointestinal diseases, by promoting the growth of beneficial gut microbiota, as well as interacting with macromolecules and having important antioxidative properties [49]. After exposure to temperature, the polyphenol concentration also decreases, while the biofortification of rye plants by applying potassium iodide to the soil resulted in a significant increase in hydrophilic and lipophilic antioxidants (glutathione, ascorbic acid, phenolic compounds) [52,53,54].

Additionally, the vitamin content is susceptible to temperature dynamics as well, with the concentration of B-vitamins being more stable in whole wheat flour than in white flour. More precisely, B1 and B6 vitamins were more susceptible to temperature changes, and their concentrations decreased significantly after the baking process, while the B2 concentration was more stable [55,56]. This effect has largely been shown in conventional baking processes. Nevertheless, due to the low concentrations of vitamins even in the raw samples, measuring the concentrations in the baked samples was not deemed necessary.

The fortification of wheat flour with minerals would lead to an increase in the nutritional value of the resulting dishes, and of bread in particular. This can also be achieved by adding flour obtained from mixing wheat flour with flour from various plants that have a high mineral content. Current cereal breeding programs are mainly focused on higher yields and higher technological quality for industrial processing, neglecting health and nutritional aspects, including possible allergenic factors that may emanate from substances used to increase productivity and control pests [57,58,59,60].

Moreover, the mineral composition of cereals is closely correlated with the chemical composition of the soil. As mentioned before, the soil in the experimental area is moderately acidic and with a low K concentration, which causes a lower assimilation of minerals in plants, mainly due to increased Al toxicity and impaired root growth and stress responses [61,62]. The organic matter consists of low levels of humus, and the low nitrogen and phosphorus contents also correlate with slowed plant growth, being strongly influenced by farming methods, with conventional ones being less harmful to the soil and environment, compared with the modern ones [63,64,65].

The low vitamin and mineral content of cereals is due to soil depletion, which is also observed in organically grown cereals where no pesticides or other chemicals are used. Diets are based mainly on processed foods from crops grown in mineral-poor soils. To increase the yield of cereals, the amount of carbohydrates is increased, and the amount of minerals and proteins is decreased. Additionally, to increase yields, but also as a result of the use of chemical fertilizers and pesticides, modern crops are harvested faster. Therefore, cereals have less time to absorb nutrients from the soil. Monoculture farming practices have also led to soil depletion, which directly affects the mineral content. Industrial development, including deforestation and exploitation, also contributes significantly to soil depletion by removing the soil layer containing the minerals needed by crops [66,67].

Consumer preferences for refined, nutrient-poor foods have led to an increase in non-communicable chronic diseases (diabetes, obesity, cardiovascular diseases). In Romania, white bread represents the most widely consumed food. Considering the low values compared to daily requirements for minerals and vitamins, several researchers have studied ways to increase these values, obtaining qualitatively superior bakery products in terms of these compounds. Depending on the chemical composition of the flour obtained from cereals, the dough and consequently the bread will have different characteristics (water absorption, protein and starch quality, amylase activity) [68].

The water retention capacity in bread after baking is over 50%, depending on the types of cereals used. Since this parameter is influenced by other factors (time, temperature, and equipment used in kneading, aspects related to the fermentation process, temperature, and humidity during baking) [69], we chose to report the results obtained after baking the dough, also per 100 g of fresh weight of the product, similar to the results obtained from the analysis of the flour from which the heat-treated dough was obtained. Taking into account the moisture retained in bread and the fact that the final results were reported per 100 g of the fresh product, the latter demonstrated a lower content of minerals compared to the flour data. These values are consistent with the results obtained by other researchers, showing a reduction in mineral levels after baking the dough. For example, in the case of Mg, the decrease is from 47.73 mg per 100 g of wheat flour to 42.16 mg per 100 g of bread, representing a decrease of 11.67% [70].

Food safety and environmental issues are important factors of increasing concern to people in both developed and developing countries. Cereals are the most widely consumed food in the world, and therefore should be an inexpensive and rich source of vitamins and minerals to reduce the incidence of chronic non-communicable diseases faced by a large part of the population regardless of age [71,72].

The limiting factor of this study is the lack of a detailed analysis of the consumption of each cereal type in terms of age, education, and presence or absence of chronic nutritional conditions. In view of the results obtained, it would be advisable to conduct a more comprehensive study of a larger area of cereal, vegetable and fruit crops throughout the country that should include the determination of the vitamin and mineral content of these foods consumed by the entire population, given that a deficiency of vitamins and minerals in the diet leads to various chronic diseases in the whole body, regardless of age. Nevertheless, to our knowledge, the existing evidence of the dynamic of the concentrations of various minerals and vitamins in cereals and legumes under temperature treatment is rather limited. Thus, we consider this research as an important foundation for further studies analyzing the matter.

## 5. Conclusions

Increasing agricultural productivity is important for food security and the supply of agricultural products to a growing world population, putting increasing pressure on food resources and soil, while altering the quality of the consumed food products. The basic grains studied were limited in their ability to provide the minerals required to meet the recommended daily intake. Following heat processing, all samples showed a decrease in mineral levels, indicating the unfavorable action of heat treatment on the nutritional quality of the products obtained. Nevertheless, soy and oat flour still recorded important mineral concentrations, and their decrease was lower than in the other samples. The content of soy flour was maintained above average even after heat treatment. Moreover, the vitamin contents, except for B6, were already below the quantification limit in the raw samples.

This study indicates the low value of micronutrients in cereals grown and harvested in the western part of Romania, and may contribute to the assessment of the measures that are meant to improve the contents of these minerals. The analysis was related to the main grain-producing area, without having reliable information on the influx of grains from other areas. Biofortification based on the application of micronutrient fertilizers can be a solution to increase the contents of micronutrients in cereals. Moreover, the study has practical implications for various stakeholders. In terms of management, it provides valuable guidance for the food industry, influencing processing techniques to enhance or fortify cereals with specific nutrients. This aligns with the growing consumer demand for healthier food options. Additionally, the results can be leveraged in consumer education initiatives, empowering individuals to make informed choices about cooking methods and their effects on nutritional value. The potential integration of study outcomes into product labeling further facilitates informed decision-making at the point of purchase.

## Figures and Tables

**Table 1:** Factor sample: mean values of cations.

Sample	Ca (mg 100 g^−1^ Sample)	Mg (mg 100 g^−1^ Sample)	Zn (mg 100 g^−1^ Sample)	Fe (mg 100 g^−1^ Sample)	Cu (mg 100 g^−1^ Sample)	K (mg 100 g^−1^ Sample)
P1	42.62 c ± 5.01	99.98 d ± 18.88	2.81 a ± 0.36	3.90 b ± 1.65	1.72 d ± 0.52	258.50 d ± 48.77
P2	22.81 d ± 4.21	49.44 e ± 14.16	1.38 c ± 0.48	1.77 c ± 0.33	0.47 e ± 0.36	102.50 e ± 6.10
P3	43.6 c ± 4.45	112.21 c ± 27.28	2.24 b ± 0.62	3.97 b ± 0.15	1.87 c ± 0.22	336.00 b ± 142.42
P4	146.20 a ± 48.43	202.60 a ± 18.2	2.415 b ± 1.33	6.83 a ± 1.90	2.18 b ± 0.24	1185.60 a ± 186.89
P5	91.90 b ± 29.52	115.60 b ± 19.83	2.99 a ± 0.14	6.61 a ± 0.55	2.36 a ± 0.29	320.30 c ± 80.3

Note: different letters that accompany the mean values denote statistically significant differences between the corresponding means. The post-hoc Dunn–Sidak test (*p* = 0.05) was used to perform multiple pairwise comparisons between mean values.

**Table 2:** Daily recommended intakes of minerals.

Mineral	Daily Requirement (mg)
K	3800
Ca	1000
Mg	420
Fe	18
Zn	14
Cu	1.7

**Table 3:** Factor Stage_Sample: mean values of cations.

Stage_Sample	Ca (mg 100 g^−1^ Sample)	Mg (mg 100 g^−1^ Sample)	Zn (mg 100 g^−1^ Sample)	Fe (mg 100 g^−1^ Sample)	Cu (mg 100 g^−1^ Sample)	K (mg 100 g^−1^ Sample)
raw_P1	46.58 f ± 4.25	117.20 h ± 1.05	3.11 c ± 0.09	5.40 f ± 0.20	2.19 e ± 0.04	303.00 g ± 2.00
raw_P2	26.62 h ± 0.48	62.35 j ± 0.85	1.80 e ± 0.10	2.05 g ± 0.15	0.80 g ± 0.07	108.00 j ± 1.50
raw_P3	47.60 f ± 0.70	137.10 g ± 1.10	2.80 d ± 0.12	4.08 e ± 0.12	2.07 d ± 0.03	466.00 h ± 2.00
raw_P4	190.40 c ± 1.10	219.20 b ± 0.80	3.63 e ± 0.07	8.56 d ± 0.16	2.39 c ± 0.06	1356.20 b ± 1.80
raw_P5	118.80 d ± 1.76	133.70 f ± 0.30	3.10 b ± 0.10	7.10 c ± 0.10	2.59 c ± 0.11	393.60 f ± 0.40
baked_P1	38.68 e ± 0.32	82.75 e ± 0.25	2.50 b ± 0.20	2.40 d ± 0.15	1.25 bc ± 0.05	214.00 e ± 1.20
baked_P2	19.00 g ± 0.70	36.52 i ± 0.48	0.95 d ± 0.15	1.48 f ± 0.08	0.140 ± 0.02	97.00 i ± 0.40
baked_P3	39.60 e ± 1.40	87.32 c ± 0.68	1.68 bc ± 0.07	3.85 e ± 0.05	1.67 c ± 0.03	206.00 c ± 1.30
baked_P4	102.00 a ± 0.70	186.00 a ± 0.80	1.20 a ± 0.10	5.10 a ± 0.20	1.96 ab ± 0.04	1015.00 a ± 2.00
baked_P5	65.00 b ± 2.00	97.50 d ± 0.70	2.89 b ± 0.06	6.12 b ± 0.18	2.12 a ± 0.18	247.00 d ± 0.70

Note: different letters that accompanies the mean values denotes statistically significant differences between the corresponding means. The post-hoc test Dunn-Sidak (*p* = 0.05) was used to perform multiple pairwise comparisons between mean values.

**Table 4:** Mean dry values of minerals in raw and baked samples.

Stage_Sample	Moisture Content (%)	Ca (mg 100 g^−1^ Sample DW)	Mg (mg 100 g^−1^ Sample DW)	Zn (mg 100 g^−1^ Sample DW)	Fe (mg 100 g^−1^ Sample DW)	Cu (mg 100 g^−1^ Sample DW)	K (mg 100 g^−1^ Sample DW)
raw_P1	10	51.76	130.22	3.46	6.00	2.43	336.67
raw_P2	12	30.30	70.95	2.05	2.32	0.91	121.74
raw_P3	8	51.74	148.91	3.04	4.15	2.12	506.52
raw_P4	7	204.60	235.59	3.90	9.20	2.57	1456.34
raw_P5	6	126.38	142.34	3.29	7.57	2.76	418.30
baked_P1	39	63.61	135.16	4.10	3.93	2.04	351.64
baked_P2	39	31.19	60.24	1.57	2.44	0.23	106.10
baked_P3	39	65.00	143.37	2.76	6.31	2.75	215.53
baked_P4	39	167.21	304.17	1.97	8.36	3.22	1673.55
baked_P5	39	106.56	159.43	4.79	10.07	3.50	255.80

DW: Dry weight.

**Table 5:** Principal component analysis (PCA) statistical results.

PC	Eigenvalue	% Variance
1	4.62	77.03
2	0.93	15.50
3	0.25	4.24
4	0.16	2.73
5	0.03	0.45
6	0.003	0.05

**Table 6:** Linear discriminant analysis (LDA) statistical results.

Axis	Eigenvalue	% Variance
1	11478	98.39
2	153.09	1.31
3	34.888	0.299

**Table 7:** Statistical significance values calculated with MANOVA (*p* = 0.05) multivariate method, from multiple pairwise comparisons of the samples (i.e., the Satge_Sample factor levels), with Bonferroni correction.

MANOVA *p*-Values	raw_P1	raw_P2	raw_P3	raw_P4	raw_P5	baked_P1	baked_P2	baked_P3	baked_P4	baked_P5
raw_P1		0.0010	0.0101	0.0001	0.0011	0.0023	0.0006	0.0053	0.0002	0.6819
raw_P2	0.0010		0.0006	0.0000	0.0003	0.0078	0.0126	0.0027	0.0001	0.0010
raw_P3	0.0101	0.0006		0.0001	0.0021	0.0012	0.0004	0.0021	0.0003	0.0111
raw_P4	0.0001	0.0000	0.0001		0.0002	0.0001	0.0000	0.0001	0.0005	0.0001
raw_P5	0.0011	0.0003	0.0021	0.0002		0.0004	0.0002	0.0005	0.0007	0.0011
baked_P1	0.0023	0.0078	0.0012	0.0001	0.0004		0.0025	0.0125	0.0001	0.0023
baked_P2	0.0006	0.0126	0.0004	0.0000	0.0002	0.0025		0.0013	0.0001	0.0006
baked_P3	0.0053	0.0027	0.0021	0.0001	0.0005	0.0125	0.0013		0.0002	0.0052
baked_P4	0.0002	0.0001	0.0003	0.0005	0.0007	0.0001	0.0001	0.0002		0.0002
baked_P5	0.6819	0.0010	0.0111	0.0001	0.0011	0.0023	0.0006	0.0052	0.0002	

**Table 8:** Mineral content of commercial white bread.

Mineral	Content (mg per 100 g)
Ca	21.2
Mg	26.83
Zn	0.84
Cu	0.12
Fe	1.62
K	103

**Table 9:** The antioxidant capacity of cereal flour before thermal processing.

Sample	Total Polyphenols	Antioxidant Capacity
	Gallic Acid Equivalent µg/g	Ascorbic Acid Equivalent µg/g
P1	1090 ± 1.63	2.34 ± 0.09
P2	154 ± 1.63	0.33 ± 0.04
P3	257 ± 3.61	0.55 ± 0.06
P4	96 ± 1.63	0.21 ± 0.01
P5	447 ± 1.63	0.96 ± 0.06

**Table 10:** The antioxidant capacity of cereal flour after thermal processing.

Sample	Total Polyphenols	Antioxidant Capacity
	Gallic Acid Equivalent µg/g	Ascorbic Acid Equivalent µg/g
P1	1079 ± 2.16	2.32 ± 0.88
P2	147 ± 1.63	0.29 ± 0.03
P3	218 ± 1.00	0.47 ± 0.04
P4	89 ± 1.00	0.19 ± 0.08
P5	433 ± 1.63	0.93 ± 0.03

**Table 11:** Vitamin content before thermal processing.

Vitamins and LQ	B1	B2	B3	B6	B12	D3	A	MK4	MK7
	0.12	0.2	0.2	0.2	0.12	0.2	0.08	0.17	0.2
Sample	mg 100 g^−1^								
P1	<LQ	<LQ	0.019	<LQ	<LQ	<LQ	<LQ	<LQ	<LQ
P2	<LQ	<LQ	0.017	<LQ	<LQ	<LQ	<LQ	<LQ	<LQ
P3	<LQ	<LQ	0.018	<LQ	<LQ	<LQ	<LQ	<LQ	<LQ
P4	<LQ	<LQ	0.019	<LQ	<LQ	<LQ	<LQ	<LQ	<LQ
P5	<LQ	<LQ	0.021	<LQ	<LQ	<LQ	<LQ	<LQ	<LQ

LQ—quantification limit.

**Table 12:** Sample characteristics.

Grain/Depth at Sowing (cm)	Sample (100 g)	Dough	Determinations
Wheat/6	P1—whole wheat flour	100 g flour + 60 g water Baking: 220 °C for 30 min in electric oven	Minerals: iron (Fe), copper (Cu), zinc (Zn), calcium (Ca), magnesium (Mg), potassium (K) Vitamins Polyphenols Antioxidant activity
	P2—hulled wheat flour		
Rye/6	P3—rye flour		
Oat/4	P5—oat flour		
Soy/4	P4—soy flour		

## Data Availability

Data are contained within the article.
